# Distinct CD4^−^CD8^−^ (Double-Negative) Memory T-Cell Subpopulations Are Associated With Indeterminate and Cardiac Clinical Forms of Chagas Disease

**DOI:** 10.3389/fimmu.2021.761795

**Published:** 2021-11-11

**Authors:** Livia Silva Araújo Passos, Carolina Cattoni Koh, Luísa Mourão Dias Magalhães, Maria do Carmo Pereira Nunes, Kenneth John Gollob, Walderez Ornelas Dutra

**Affiliations:** ^1^ Departamento de Morfologia Instituto de Ciências Biológicas, Universidade Federal de Minas Gerais, Belo Horizonte, Brazil; ^2^ Pós-graduação em Parasitologia, Instituto de Ciências Biológicas, Universidade Federal de Minas Gerais, Belo Horizonte, Brazil; ^3^ Departamento de Clínica Médica, Faculdade de Medicina, Universidade Federal de Minas Gerais, Belo Horizonte, Brazil; ^4^ Hospital Israelita Albert Einstein, São Paulo, Brazil; ^5^ Instituto Nacional de Ciência e Tecnologia Doenças Tropicais—INCT-DT, Belo Horizonte, Brazil

**Keywords:** *Trypanosoma cruzi*, pathology, immunoregulation, Chagas disease, cardiomyopathy, memory response, double-negative T cells

## Abstract

CD4^−^CD8^−^ (double-negative, DN) T cells are critical orchestrators of the cytokine network associated with the pathogenic inflammatory response in one of the deadliest cardiomyopathies known, Chagas heart disease, which is caused by *Trypanosoma cruzi* infection. Here, studying the distribution, activation status, and cytokine expression of memory DN T-cell subpopulations in Chagas disease patients without cardiac involvement (indeterminate form—IND) or with Chagas cardiomyopathy (CARD), we report that while IND patients displayed a higher frequency of central memory, CARD had a high frequency of effector memory DN T cells. In addition, central memory DN T cells from IND displayed a balanced cytokine profile, characterized by the concomitant expression of IFN-γ and IL-10, which was not observed in effector memory DN T cells from CARD. Supporting potential clinical relevance, we found that the frequency of central memory DN T cells was associated with indicators of better ventricular function, while the frequency of effector memory DN T cells was not. Importantly, decreasing CD1d-mediated activation of DN T cells led to an increase in IL-10 expression by effector memory DN T cells from CARD, restoring a balanced profile similar to that observed in the protective central memory DN T cells. Targeting the activation of effector memory DN T cells may emerge as a strategy to control inflammation in Chagas cardiomyopathy and potentially in other inflammatory diseases where these cells play a key role.

## Introduction

The generation and maintenance of immunological memory is fundamental to preserve protective immune responses capable of controlling pathogens and of immune surveillance (1, 2). Memory T-cell differentiation progresses from naive cells toward central memory (CM) and effector memory (EM) subpopulations (3). While CM cells, defined as CD45RA^–^CCR7^+^, must migrate into secondary lymphoid organs (SLO) and undergo recall stimulation before becoming fully functional, EM cells, defined as CD45RA^–^CCR7^–^, can migrate directly to the target tissue and perform effector functions (4–6). Despite these differences in migration and activation requirements, both subpopulations execute effector responses and produce cytokines (7, 8). Effector cells (EF, CD45RA^+^CCR7^−^), generated right after activation of naive cells, can also perform effector functions without the need to transit through SLO but, as opposed to CM and EM, are typically short-lived (9).

CM, EM, and EF subpopulations have been identified in CD4^+^ and CD8^+^ T cells, exhibiting similar characteristics (7, 10). Double-negative (CD4^−^CD8^−^, DN) T cells comprise approximately 3% of the total human circulating lymphocytes and can express αβ or γδ T-cell receptors (TCR) (11). For the lack of co-receptors, they tolerate chronic stimulation, rendering them crucial in immunity to chronic infections (12). DN T cells are typically activated by glycoconjugates and lipids presented by CD1 molecules (13, 14). Protective and pathogenic immune responses in human diseases have been associated with DN T cells (13, 15–17), including those caused by parasites, which are adorned with highly immunogenic glycoconjugates (18–20).

The functional characteristics of DN T cells have been studied in patients with Chagas disease (13, 18, 21), which is a long-lasting chronic infection caused by the protozoan *Trypanosoma cruzi*. The vast majority of Chagas disease patients do not present clinical signs, classified as indeterminate (22), while at least 30% progress to Chagas cardiomyopathy (22, 23). The cardiomyopathy is caused by an inflammatory reaction that leads to heart failure and death (24, 25). There are no vaccines to prevent infection and no effective therapies exist to halt disease progression. Finding strategies to prevent pathology development and progression is a pressing need.

Previous studies by our group have shown that DN T cells from indeterminate and cardiac Chagas patients are robust producers of inflammatory and anti-inflammatory cytokines and that the balance of these cytokines is shifted toward an anti-inflammatory or inflammatory profile, in indeterminate and cardiac patients, respectively (19). In addition, we showed that *T. cruzi*-derived glycolipids trigger activation of DN T cells from both indeterminate and cardiac patients, and the resulting proinflammatory response observed in cardiac Chagas patients can be minimized by blocking this activation *in vitro* (13, 18, 19, 26). Thus, DN T cells are potential targets to diminish inflammation in Chagas cardiomyopathy. Here, we employed multiparameter flow cytometry to determine the distribution, activation, and cytokine profile of DN T cells from indeterminate and cardiac patients with Chagas disease. In addition, we performed *in vitro* blocking assays to show that manipulation of DN T-cell activation can be a useful strategy to modify EM cells toward a less inflammatory profile in Chagas cardiomyopathy patients. The ability to manipulate EM DN T cells may also have implications in other chronic inflammatory diseases where these cells play a role.

## Patients, Material, and Methods

### Patient Population

A total of 18 patients with indeterminate (*n* = 6) or dilated cardiomyopathy (*n* = 12) clinical forms of Chagas disease were enrolled. Serology exams for Chagas disease, electrocardiogram, and chest X-rays were performed to define the clinical status of the patient (27). Indeterminate patients (IND) had positive serology and lacked evidence of digestive or cardiac pathology and the absence of clinical signs and symptoms. Chagas cardiomyopathy patients (CARD) displayed positive serology and lacked evidence of digestive pathology, with some degree of myocardium damage expressed by abnormal electrocardiogram. Left ventricular ejection fraction (LVEF) and left ventricular diastolic diameter (LVDD) were used as parameters of disease severity (27). Chronic inflammatory diseases, diabetes, heart/circulatory illnesses, or bacterial infections were used as exclusion criteria. **Table 1** summarizes the clinical characteristics of the patients. The patients did not previously receive specific treatment for Chagas disease.

**Table 1:** Chagas disease patients enrolled in the study.

Patient ID	Clinical form	Age (years)	Gender	LVDD (mm)	LVEF (%)
I1	Indeterminate	57	Male	42	73
I2	Indeterminate	45	Female	47	66
I3	Indeterminate	45	Female	51	65
I4	Indeterminate	43	Female	44	76
I5	Indeterminate	52	Female	46	74
I6	Indeterminate	61	Female	47	62
D1	Cardiac	48	Male	57	20
D2	Cardiac	56	Female	55	36
D3	Cardiac	41	Male	52	56
D4	Cardiac	48	Male	59	45
D5	Cardiac	39	Female	70	34
D6	Cardiac	60	Female	40	54
D7	Cardiac	–	Male	–	–
D8	Cardiac	69	Female	54	45
D9	Cardiac	42	Male	–	–
D10	Cardiac	49	Male	69	34
D11	Cardiac	–	Female	60	55
D12	Cardiac	67	Male	–	–

All patients were from Minas Gerais State, Brazil.

This study was approved by the Ethical Committee from the Federal University of Minas Gerais (COEP-UFMG–2.809.859) and complied with the Helsinki Declaration. Treatment and clinical care were offered to all volunteers, and all signed the informed consent form prior to inclusion in the study.

### Parasites

Trypomastigotes (TRPs) of the Y strain of *T. cruzi* were grown in Vero cells (28). Cells were infected with 10 TRPs/cell, cultured in RPMI with 5% fetal calf serum (FCS) and antibiotic (penicillin 500 U/ml and streptomycin 0.5 mg/ml) for 6 days. Cultures were carried out in an incubator at 37°C, 5% CO_2_. TRPs were collected for immediate infection of the peripheral blood mononuclear cells (PBMC) of the patients. TRPs were also used to prepare soluble antigens (18, 21), to be used in CD1d blocking assays.

### Blood Sampling and *In Vitro* Infection

Heparinized peripheral blood samples were collected and PBMCs were obtained (13, 18). PBMCs, resuspended at a concentration of 10^7^ cells/ml, were infected using a ratio of 10 parasites/cell, incubated at 37°C in 5% CO_2_ for 3 h, and washed for the removal of free TRPs. RPMI supplemented with 5% heat-inactivated human serum (Sigma-Aldrich, St. Louis, MO, USA), antibiotics (penicillin, 200 U/ml; and streptomycin, 0.1 mg/ml) (Sigma-Aldrich, St. Louis, MO, USA), and L-glutamine (1 mM) (Sigma-Aldrich, St. Louis, MO, USA) was added to a final volume of 1 ml. Infected PBMCs were reincubated at 37°C in 5% CO_2_ for 14 h. Brefeldin A (1 μg/ml, Sigma-Aldrich, St. Louis, MO, USA) was added, and cultures were reincubated for an additional 4 h. Non-stimulated controls (MED) were included.

For the blocking experiments, 3 × 10^5^ PBMCs were incubated with anti-CD1d antibodies (25 μg/ml) and 20 μg/ml of TRP antigens (TRP-SA). Cultures were performed as described above and included PBMCs incubated with media only, media plus anti-CD1d, TRP-SA only, and TRP-SA plus anti-CD1d.

### Cell-Surface Phenotype and Intracellular Cytokine Staining Using Flow Cytometry

Combinations of monoclonal antibodies (mAbs) anti-CD4, anti-CD8, anti-TCR αβ, anti-TCR γδ, anti-CCR7, anti-CD45RA, and anti-CD69 were used to determine circulating T memory subsets in DN T cells and their activation state by multiparametric flow cytometry. CD69 is a molecule often used as an activation marker for T cells and has previously been used by our group, studying Chagas disease (19, 20). Antibodies were added to each tube containing 5 × 10^5^ cells (concentration determined using trypan blue stain), for 20 min at 4°C. Samples were washed in PBS containing 1% bovine serum albumin (BSA) and fixed for 20 min with 2% formaldehyde solution. After washing with PBS, cells were permeabilized for 15 min with a 0.5% saponin solution and proceeded to intracellular staining. Samples were incubated with anti-IL-10 and/or anti-IFN-γ for 20 min at room temperature, washed twice with 0.5% saponin solution, resuspended in PBS, and read in FACSCanto II (Becton Dickenson, San Jose, CA, USA). There were 100,000 cells/sample that were collected. FACS data were analyzed using FlowJo (Becton Dickenson, San Jose, CA, USA), using exclusion of doublets for all samples. Positive cells were selected based on isotype controls to set the negatives. T-distributed stochastic neighbor-embedding (t-SNE) unsupervised analysis was performed to segregate cell populations and determine population trajectory analysis. All antibodies were from BioLegend (San Diego, CA, USA) (**Supplementary Table 1**).

### Statistical Analysis

All data showed a Gaussian distribution, as determined by the Kolmogorov–Smirnov test. Paired *t*-test was used to compare unstimulated *versus* stimulated cultures within the same group of patients. Unpaired *t*-test was used to compare data between different groups of patients (for example, non-stimulated cultures from IND *versus* CARD, or stimulated cultures from IND *versus* CARD). Correlation analyses were performed using Pearson’s coefficient. *p*-values ≤0.05 were considered statistically significant. Heatmap analysis was performed using the ClustVis software package R-version 0.7.7 (Metsalu, Tauno and Vilo, Jaak. ClustVis: a web tool for visualizing clustering of multivariate data using Principal Component Analysis and heatmap. Nucleic Acids Research, 43(W1):W566–W570, 2015. doi: 10.1093/nar/gkv468).

## Results

### Indeterminate Patients Display Higher Percentages of DN T Cells With Central Memory and Naive Phenotypes, Whereas Cardiomyopathy Patients Display Higher Percentages of Effector Memory and Effector DN T Cells

**Figure 1:**
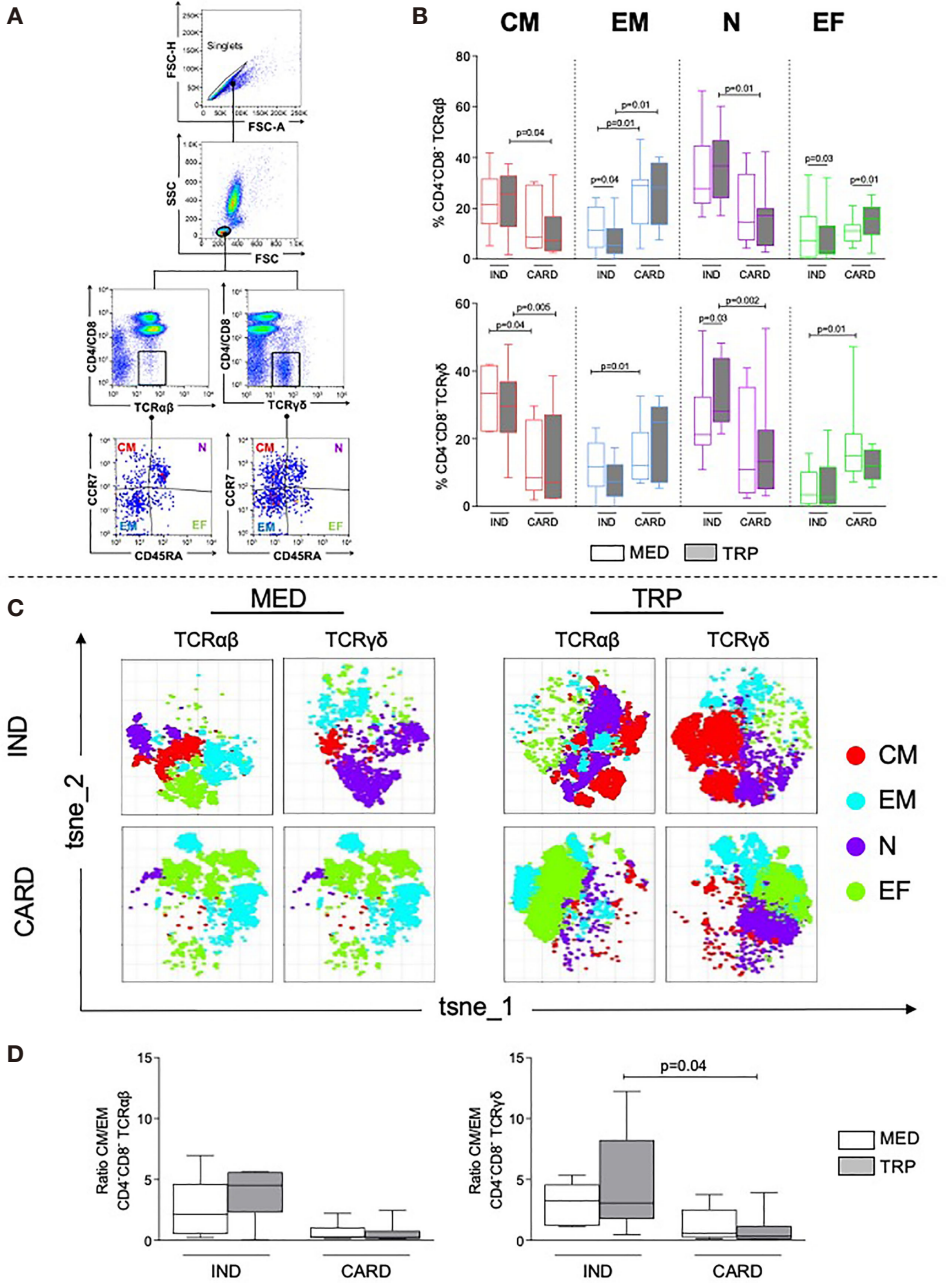
Analysis of double-negative (DN) memory subpopulations, naive and effector cells in indeterminate (IND, *n* = 6) and cardiac (CARD, *n* = 6) Chagas patients. **(A)** Representative dot plots illustrating the selection of DN T-cell subpopulations after selection of singlets: central memory (CM), effector memory (EM), naive (N), and effector cells (EF). Representative plots were performed using data from one IND patient, in non-stimulated culture. **(B)** Frequencies of DN TCR αβ^+^ or γδ^+^ CM, EM, N, and EF cells before (MED) and after *in vitro* stimulation with live parasite (TRP). **(C)** t-SNE from combined data obtained from all IND and CARD patients using MED or TRP cultures, in TCR αβ^+^ or TCR γδ^+^ DN T cells. Colors correspond to phonograph-guided clustering. **(D)** Ratio of CM/EM DN T-cell memory subpopulations in IND and CARD, before (MED) and after *in vitro* stimulation with live parasite (TRP). Results in **(B, D)** are expressed as percentage in box plot, extending from the 25th to 75th percentile, with a horizontal line at the median with whiskers. Paired or unpaired *t*-tests were used to compare unstimulated and stimulated cultures or cultures between different groups of patients, respectively. Statistical significance is indicated in each graph.

The gating strategy to determine the distribution of central memory (CD45RA^–^CCR7^+^), effector memory (CD45RA^–^CCR7^–^), naive (CD45RA^+^CCR7^+^), and effector (CD45RA^+^CCR7^−^) subpopulations is shown in **Figure 1A**.

IND displayed a higher percentage of central memory and naive DN T cells in TCR αβ^+^ and TCR γδ^+^ subpopulations as compared with CARD, especially after TRP stimulation (**Figure 1B**). However, CARD had a higher percentage of effector memory DN T cells in TCR αβ^+^ and TCR γδ^+^ subpopulations in non-stimulated cultures, which was also more evident after TRP stimulation (**Figure 1B**). The frequency of effector cells was higher in TCR γδ^+^ DN T cells from CARD as compared with IND (**Figure 1B** and **Supplementary Figure 3B**).

Unsupervised t-SNE analysis clearly confirmed the predominance of central memory and naive in IND and of effector memory and effector DN T cells in CARD, especially after parasite stimulation (**Figure 1C**). We observed a higher central/effector memory ratio in IND as compared with CARD, which was statistically significant in TCR γδ^+^ cells after TRP stimulation (**Figure 1D**).

### Co-Expression of IL-10 and IFN-γ Is Higher in Central Memory DN T Cells From Indeterminate Patients

**Figure 2:**
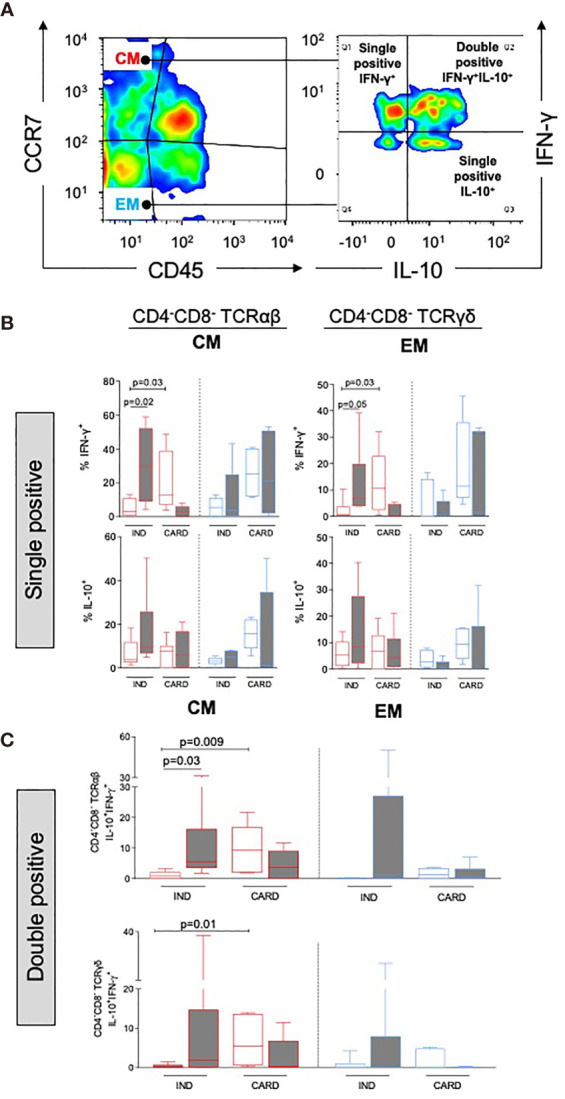
Analysis of cytokine expression in DN memory subpopulations from indeterminate (IND, *n* = 6) and cardiac (CARD, *n* = 6) Chagas patients. **(A)** Representative density color illustrating the distribution of DN T-cell subpopulations and selection of CM DN T cells, followed by analysis of single and double-positive IFN-γ^+^ and/or IL-10^+^ cells. Representative density color was performed using data from one IND patient, after *in vitro* stimulation with live parasite (TRP). **(B)** Frequency of IFN-γ^+^ or IL-10^+^ in central memory (CM) and effector memory (EM) in DN TCR αβ and DN TCR γδ subpopulations. **(C)** Frequency of cells co-expressing IFN-γ^+^ and IL-10^+^ in CM and EM in DN TCR αβ and DN TCR γδ subpopulations. The results are expressed as percentage in box plot, extending from the 25th to 75th percentile, with a horizontal line at the median with whiskers. Paired or unpaired *t*-tests were used to compare unstimulated and stimulated cultures or cultures between different groups of patients, respectively. Statistical significance is indicated in each graph.

Central memory TCR αβ^+^ and γδ^+^ DN T cells from CARD displayed a higher expression of IFN-γ before stimulation as compared with IND. TRP stimulation increased IFN-γ production by central memory cells from IND (**Figure 2A**, top). Although without statistical significance, effector memory cells from CARD showed a clear tendency of higher expression of IFN-γ as compared with IND (**Figure 2B**, top panel; **Supplementary Figure 4B**, top panel). No differences were observed regarding IL-10 expression (**Figure 2B**, bottom panel; **Supplementary Figure 4B**, bottom panel). TRPs lead to an increase in the frequency of IL-10^+^IFN-γ^+^ DN T cells in central memory TCR αβ^+^ subpopulation from IND (**Figure 2C** and **Supplementary Figure 4C**).

### The Activation Marker CD69 Is Differentially Expressed in DN T-Cell Memory Populations From Indeterminate and Chagas Cardiomyopathy Patients

**Figure 3:**
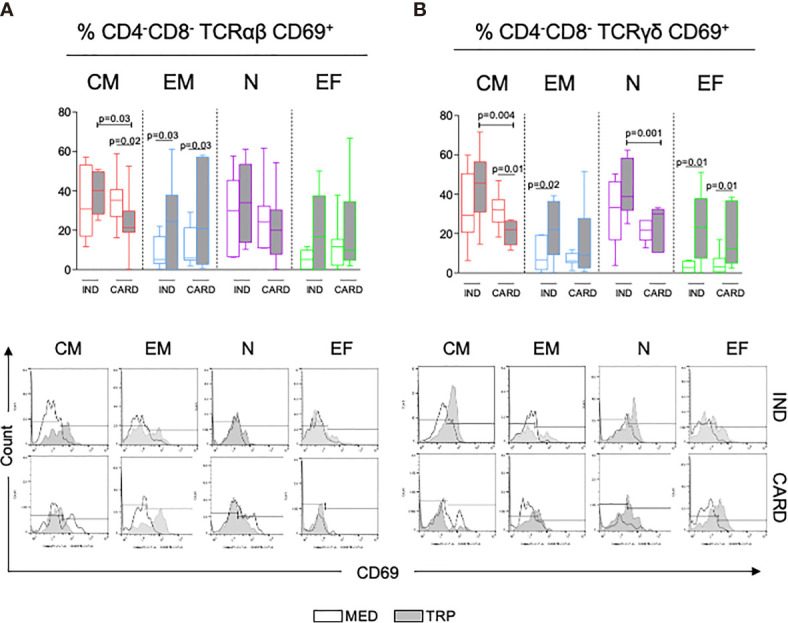
Analysis of activation status of DN T-cell central (CM) and effector (EM) memory subpopulations, naive (N) and effector (EF) cells in indeterminate (IND, *n* = 6) and cardiac (CARD, *n* = 6) Chagas patients gauged by CD69 expression, before (MED) and after *in vitro* stimulation with live parasite (TRP). The graphs (**A**, **B**, top) represent the frequency of activated DN T-cell subpopulations in the different groups and under distinct conditions. The results are expressed as percentage in box plot, extending from the 25th to 75th percentile, with a horizontal line at the median with whiskers. Paired or unpaired *t*-tests were used to compare unstimulated and stimulated cultures or cultures between different groups of patients, respectively. Statistical significance is indicated in each graph. (**A, B**, bottom panels) Representative histograms of the expression of CD69 in the different DN T-cell subpopulations in IND and CARD. Clear curves show CD69 expresion in MED and gray curves show CD69 expression in TRP.

Central memory DN T cells TCR αβ^+^ and γδ^+^ displayed a higher expression of CD69 in IND as compared with CARD (**Figures 3A, B** and **Supplementary Figures 5A, B**), especially after TRP stimulation. TRP stimulation decreased the frequency of central memory CD69^+^ cells in TCR α/β and γ/δ subpopulations from CARD (**Figures 3A, B** and **Supplementary Figures 5A, B**), while it increased the expression of CD69 in TCR αβ^+^ effector memory DN T cells from IND and CARD (**Figure 3A** and **Supplementary Figure 5A**). Although no changes in CD69 expression by naive TCR αβ^+^ cells were observed in IND, TCR γδ^+^ naive cells expressed a higher percentage of CD69 after TRP stimulation in IND than in CARD (**Figure 3B** and **Supplementary Figure 5B**). No differences were observed in CD69 expression by TCR αβ^+^ effector DN T cells between IND and CARD before or after stimulation. However, TRP stimulation increased CD69 in TCR γδ^+^ effector DN T cells in IND and CARD (**Figure 3B** and **Supplementary Figure 5B**).

t-SNE analysis highlights the differential expression of CD69 by αβ^+^ central memory DN T cells from IND and CARD after TRP stimulation (**Supplementary Figure 1A**). CD69 expression co-localizes with IL-10 in IND, while a decrease in these two markers is evident in CARD.

### Cluster Analysis of Central and Effector Memory DN T Cells Segregates Indeterminate and Chagas Cardiomyopathy Patients

**Figure 4:**
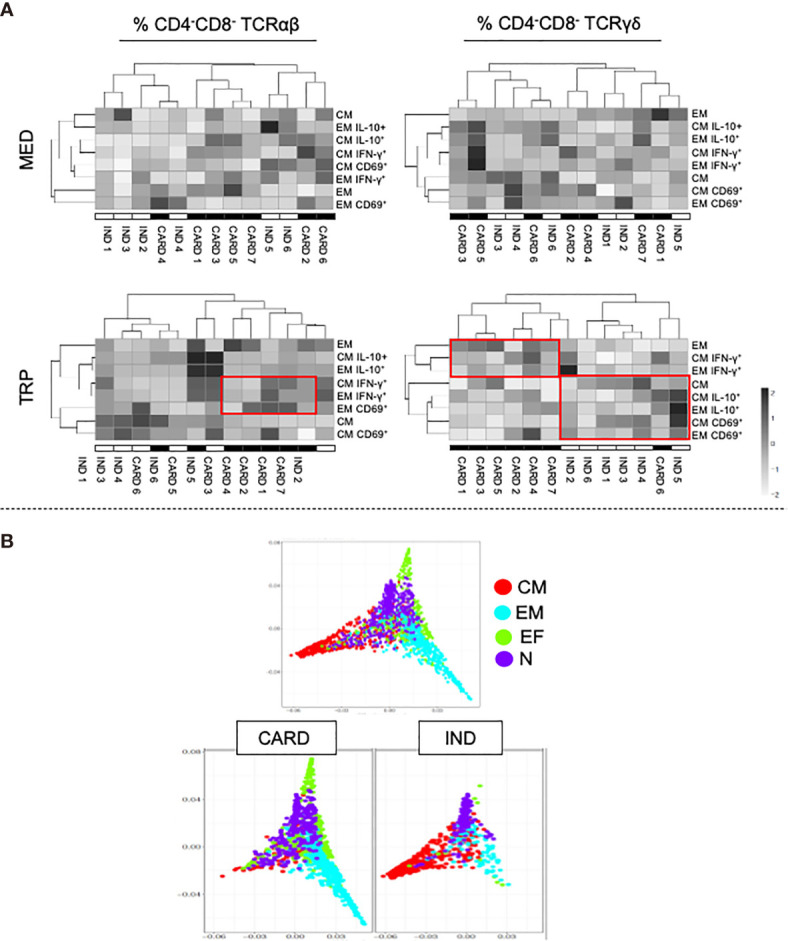
Cluster heatmap analysis of activation marker (CD69) and cytokine expression (IFN-γ and IL-10) in DN T-cell subpopulations from indeterminate (IND) and cardiac (CARD) Chagas patients. **(A)** Top panels contain the cluster heatmap analysis of non-stimulated cultures (MED) and bottom panels contain the cluster heatmap analysis after *in vitro* stimulation with live parasite (TRP). The left panels show the distribution of TCR αβ DN T cells, and the right panels the TCR γδ DN T cells. **(B)** Difusion maps showing the trajectories and relationships of the different DN T-cell subpopulations. The top plots contain all patients grouped together and the bottom plots discriminate IND and CARD, showing distinct trajectories progressing toward EM and CM, respectively.

We performed cluster analysis using the frequencies of central and effector memory DN T cells and their expression of cytokines and activation markers. While the analysis in unstimulated cells did not allow for a clear separation between IND and CARD, TRP stimulation did (**Figure 4A**). Segregation was more evident in γδ^+^ cells, showing IND associated with the frequency of central memory cells and IL-10 and CD69 expression, while CARD was associated with the frequency of effector memory cells and IFN-γ expression (**Figure 4A**). The trajectory plot (**Figure 4B**, top) shows the overall diversion of central and effector memory from the main naive population. When we segregated the distribution into IND and CARD (bottom plots), we observed a clear progression toward effector memory in CARD and toward central memory in IND, confirming the association of the clinical outcomes with different memory populations.

### Central Memory and Effector Memory DN T Cells Are Differentially Correlated With Ventricular Cardiac Function in Chagas Disease Patients

**Figure 5:**
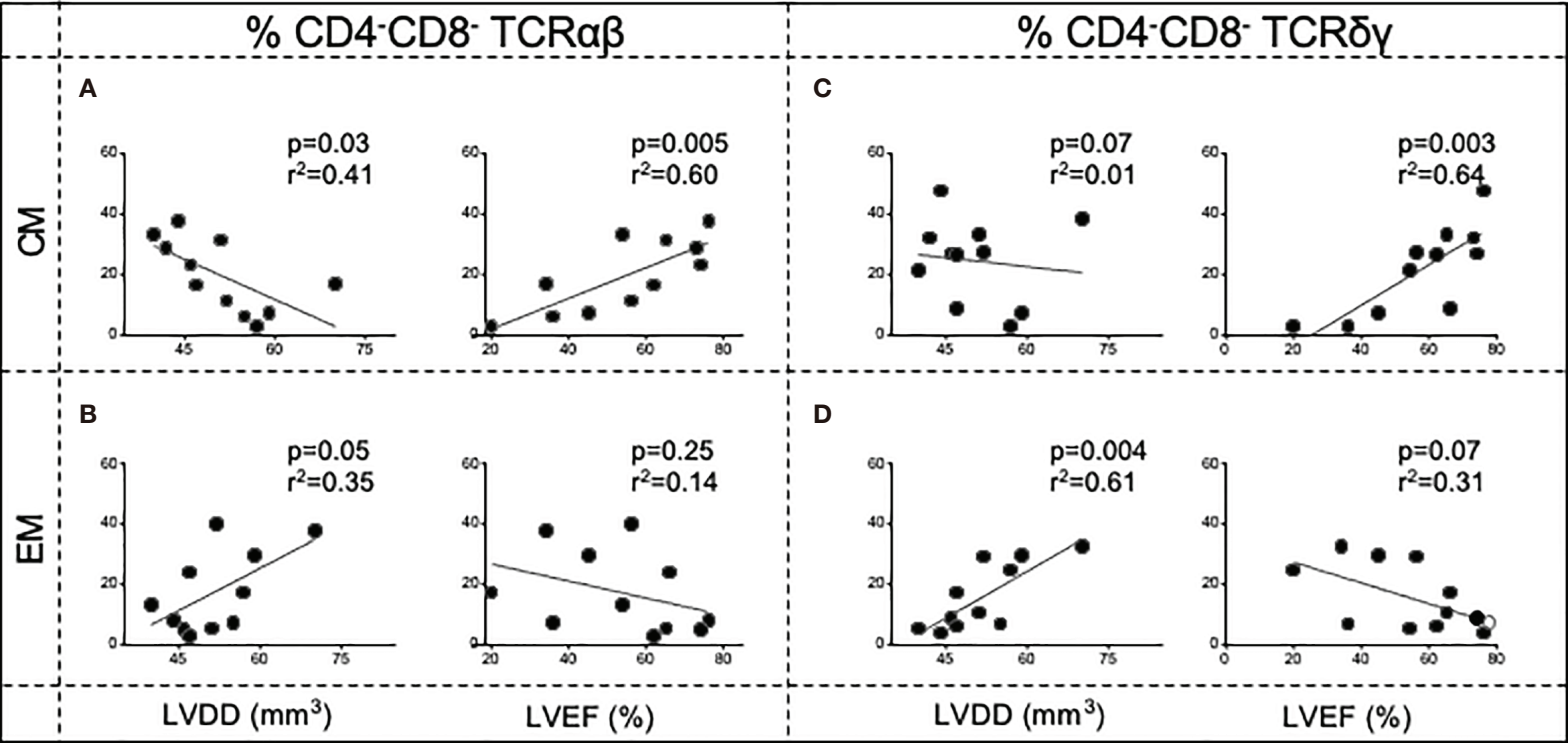
Correlation analysis of the frequency of DN central memory cells (CM—**A, C**) and DN effector memory cells (EM—**B, D**) from Chagas patients (*n* = 11) and clinical parameters of disease severity: left ventricular ejection fraction (LVEF) and left ventricular diastolic diameter (LVDD). The left panels contain the correlation analysis in TCR αβ DN T cells, and the right panels contain the correlation analysis in TCR γδ DN T cells. Correlation analyses were performed using Pearson’s coefficient. *p*-values and *r* ^2^ are indicated in each graph.

A higher frequency of central memory DN TCR αβ^+^ subpopulation was negatively correlated with LVDD and positively correlated with the LVEF, parameters related to the degree of the left ventricular dilatation and ventricular ejection function, respectively (**Figure 5A**). A similar trend was observed for the DN TCR γδ^+^ cells, with a significant positive correlation with LVEF (**Figure 5C**). Opposingly, the frequency of effector memory DN TCR αβ^+^ and γδ^+^ cells was positively correlated with LVDD (**Figures 5B, D**), displaying a trend of negative association with LVEF (**Figures 5B, D**). These data reinforce the association between central memory DN T cells with protective responses and better cardiac function and effector memory DN T cells with impairment of cardiac function in Chagas disease.

### Blocking CD1d-Mediated Activation Is Associated With Increase of IL-10 Expression by Effector Memory Cells From Patients With Severe Cardiomyopathy

**Figure 6:**
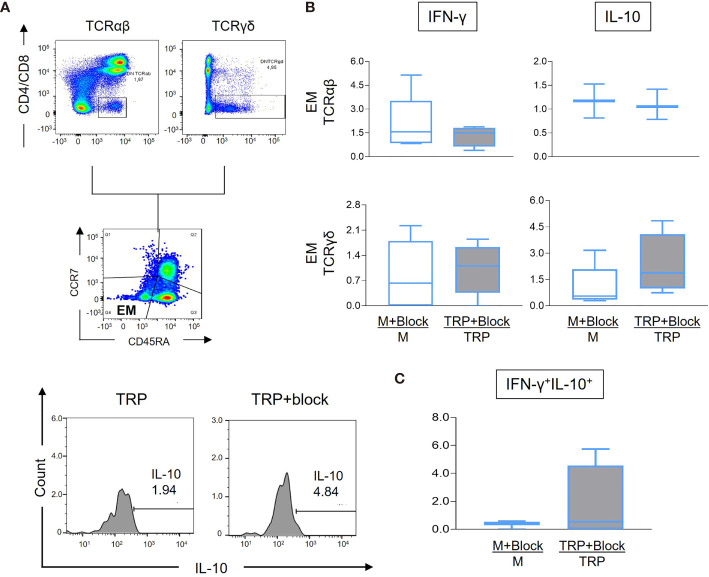
Analysis of IFN-γ and IL-10 expression by effector memory (EM) DN T-cell subpopulations from cardiac Chagas patients (CARD, *n* = 4) before and after treatment with anti-CD1d monoclonal antibodies, as described in the *Material and Methods*. **(A)** Representative FACS plots showing the gating of CD4^−^CD8^−^ TCR αβ^+^ or TCR γδ^+^ EM cells, followed by histograms of IL-10 expression measured in the presence of media alone (M), media plus anti-CD1d monoclonal antibody (M+block), parasite antigen alone (TRP-SA), or presence of TRP-SA plus anti-CD1d monoclonal antibody (TRP-SA+block). Representative plots were performed using data from one CARD patient, in stimulated culture. Histograms were from cultures stimulated and treated with blocking antibody. **(B)** Percent frequency of TCR αβ^+^ or TCR γδ^+^ EM DN T cells expressing IFN-γ and IL-10 as indicated. **(C)** Frequency of TCR γδ^+^ EM DN T cells co-expressing IFN-γ and IL-10. The results are expressed as percentage ratio for each culture condition (media+block/media or TRP-SA+block/TRP-SA) in box plots, extending from the 25th to 75th percentile, with a horizontal line at the median with whiskers. Paired *t*-tests were used to compare unstimulated and stimulated cultures. Statistical significance is indicated in the graph.

Given that we determined that effector memory DN T cells are predominant in CARD, associated with a more inflammatory profile and worse cardiac function, we sought to investigate if reducing antigen presentation *via* blocking the CD1d molecule would interfere with the cytokine profile of effector memory cells from CARD. **Figure 6A** shows representative gating strategy for the analysis. Although we did not observe statistically significant changes in IFN-γ expression in effector memory cells after CD1d blocking comparing stimulated and non-stimulated cultures (**Figure 6B**), CD1d blocking led to a statistically significant increase in IL-10 expression in γδ^+^ effector memory cells in stimulated cultures (**Figure 6B**). No statistically significant changes were observed in the frequency of central memory, effector, and naive DN T cells expressing IFN-γ or IL-10 (**Supplementary Figure 2**). Although the result did not reach statistical significance, the frequency of IFN-γ^+^IL-10^+^ also increased in the TCR γδ^+^ effector memory subpopulations after CD1d blocking, suggesting the establishment of a more balanced, potentially protective profile (**Figure 6C**).

## Discussion

Here, we show that human DN T-cell subpopulations display a distinct distribution in IND and CARD Chagas patients. While IND has a higher frequency of CM, CARD has a higher percentage of circulating effector memory (EM) DN T cells. These CM and EM DN T cells differ, in both clinical forms, as to the expression of the activation marker CD69 and the cytokines IFN-γ and IL-10, allowing to segregate IND and CARD using cluster analysis. CM DN T cells display a more balanced cytokine profile in IND than CARD, with a higher frequency of IFN-γ^+^IL-10^+^ cells. Moreover, a higher frequency of CM DN T cells is correlated with echocardiographic parameters of ventricular function, suggesting a protective role for these cells. Importantly, we show that manipulating the activation of DN T cells from CARD by inhibiting CD1d-mediated antigen presentation affects EM DN T cells, predominant in these patients, rendering them less inflammatory by increasing IL-10 expression and leading to a more balanced profile. Together, these results show that it is possible to manipulate the activation of EM DN T cells in patients with severe Chagas cardiomyopathy, reverting their cytokine profile to the one observed in the protective CM DN T cells from IND.

The subpopulations of systemic memory T cells can be subdivided into CM and EM distinguished primarily by their ability to perform effector function and expression of homing receptors (7). CM cells constitutively express CCR7, CD45RA, and CD62 and produce IL-4 and IFN-γ in response to antigen stimulation. EM cells cease to express CCR7 and CD45RA and may or may not express CD62L, displaying a faster response to antigens, high cytotoxic potential, and cytokine production (7). Thus, progression from CM to EM is accompanied by changes in phenotypic and, importantly, in functional capacities. Our data suggest that this progression in Chagas patients may lead to the loss of a protective response, executed by CM T cells, and the establishment of a pathogenic response, due to the activity of EM T cells. Our results of the trajectory analysis indeed suggest that cells from CARD progress to the EM profile, while DN T cells from IND remain with the CM phenotype. Hence, manipulating the profile of EM cells in CARD may be the key to maintain a favorable immune response.

The frequency and functional characteristics of CM and EM DN T cells allowed to segregate IND and CARD in a cluster analysis, which was particularly evident within TCR γδ^+^ DN T cells. Our previous studies of DN T cells in Chagas disease suggested that the TCR γδ^+^ subpopulation is more responsive than the αβ^+^ counterpart (13, 19), which was confirmed here for the different memory populations, especially the EM cells. TCR γδ^+^ DN T cells display intense cytotoxic activity to viral and bacterial infections (29, 30) and are associated with oligoclonal antigen recognition (29). We have demonstrated that TCR γδ^+^ DN T cells from Chagas disease patients express higher levels of activation markers upon stimulation with *T. cruzi*-derived glycoconjugates than do TCR αβ^+^ DN T cells (18). Pinpointing the antigen responsible for the activation of DN T cells emerges as a strategy to further modulate the activation of these cells, potentially interfering with the immune response to establish (or re-establish) a protective profile. The fact that CD1 molecules that present antigens to DN T cells are not polymorphic, as opposed to MHC molecules, may also present an advantage in discovering the stimulating antigen.

Our results that DN T cells of the CM phenotype are predominant and associated with a more balanced immune response in IND agree with studies evaluating the memory response of CD4^+^ T cells in human Chagas disease (31). Those studies also suggested that the CM response in the CD8^+^ T-cell compartment was associated with a higher expression of IFN-γ in CARD, but not IND. Opposingly, it has been shown that the frequency of CD8^+^ EM T cells is higher in IND than in CARD and that the expression of IFN-γ was lower in CD8^+^ T cells from CARD, suggesting that CD8^+^ T cells undergo exhaustion in the latter (32). It is important to emphasize that the lack of co-receptor renders DN T cells more resistant to chronic stimulation and possibly exhaustion (12), highlighting the importance of these cells as potential targets of immune modulation in Chagas disease.

Our data showed that CM DN T cells display a more balanced cytokine profile in IND, given the increased frequency of IFN-γ^+^IL-10^+^ cells, which was lower in CARD. The self-regulatory capacity of IFN-γ-producing cells by concomitant expression of IL-10 has been demonstrated, highlighting its clinical implications (33, 34). Jankovic et al. showed that IFN-γ-producing Th1 cells were the main source of host-protective IL-10 in infection with *Toxoplasma gondii* (35). While IFN-γ was important to control the parasite, the concomitant expression of IL-10 avoided tissue destruction. The co-expression of IFN-γ and IL-10 by CD4^+^ T cells has been associated with protective responses induced by vaccination in experimental infection with *T. cruzi* (36). Also, in autoimmune diseases and recovery after inflammatory injury, the co-expression of IFN-γ and IL-10 has been associated with favorable responses (37–39). Despite the clear trend in increase of IFN-γ^+^IL-10^+^ EM cells after blocking of CD1d, these analyses were not statistically significant, possibly due to the fact that only four patients were included in these experiments. In addition, including non-specific antibodies would provide information regarding the specific effects of the anti-CD1d antibody. Thus, future studies will be performed to tackle these limitations and to confirm if controlling DN T-cell activation increases the frequency of IFN-γ^+^IL-10^+^ EM cells in CARD, suggesting that modulating these cells renders them more similar to CM DN T cells. In our previous work characterizing the DN T cells in Chagas disease (19), we compared the profile of those cells from patients with the indeterminate and cardiac forms of Chagas disease and non-Chagas individuals. We observed that the DN T cells from non-Chagas patients did not respond to parasite antigens, as measured by the expression of activation markers and inflammatory and anti-inflammatory cytokines. In the current study, given those results and since we were focused on memory cells and recall responses, we did not include non-Chagas individuals.

Given the fact that DN T cells are major producers of inflammatory cytokines in CARD (19) and their high frequency of EM DN T cells, demonstrating that it is possible to alter the EM DN T-cell inflammatory profile in CARD opens possibilities to interfere with the immune response of the patient to control pathology. Importantly, the lack of alteration on the expression of IFN-γ and IL-10 by central memory and effector, as well as naive DN T-cell subpopulations, infers that their immune function was not affected by the blocking of activation using anti-CD1d. Our data also have implications in other diseases where DN T cells play a role in pathology, as well as in the establishment of protective memory responses.

## Data Availability Statement

The original contributions presented in the study are included in the article/**Supplementary Material**. Further inquiries can be directed to the corresponding author.

## Ethics Statement

The studies involving human participants were reviewed and approved by the Ethical Committee from the Federal University of Minas Gerais (COEP-UFMG–2.809.859). The patients/participants provided their written informed consent to participate in this study.

## Author Contributions

LP, CK, and LM performed all cell processing and cultures as well as FACS analysis. LP and CK also contributed to the study design and analysis. MN was responsible for clinical care, characterization, and material collection from all patients. KG contributed to the experimental design and data analysis. WD designed the studies and overlooked all experiments and data analysis. All authors contributed to the article and approved the submitted version.

## Funding

This work was supported by FAPEMIG (#Universal 2014), CNPq (#Universal 2015), INCT-DT, and NIAID-NIH (1R01AI138230-01). CK, MN, KG, and WD are CNPq fellows and LM is a CAPES fellow.

## Conflict of Interest

The authors declare that the research was conducted in the absence of any commercial or financial relationships that could be construed as a potential conflict of interest.

## Publisher’s Note

All claims expressed in this article are solely those of the authors and do not necessarily represent those of their affiliated organizations, or those of the publisher, the editors and the reviewers. Any product that may be evaluated in this article, or claim that may be made by its manufacturer, is not guaranteed or endorsed by the publisher.
