# Supplementary material for: Distinct CD4−CD8− (Double-Negative) Memory T-Cell Subpopulations Are Associated With Indeterminate and Cardiac Clinical Forms of Chagas Disease
Source: Front Immunol. 2021 Nov 11;12:761795. doi: 10.3389/fimmu.2021.761795 (PMC8632628; doi:10.3389/fimmu.2021.761795)
Supplement: Supplementary file 1 [file DataSheet_1.docx]

**Supplementary figure 1:** Analysis of CD69 and IL-10 expression in DN T-cell central (CM) and effector (EM) memory subpopulations, naïve (N) and effector (EF) cells in indeterminate (IND; n=6) and cardiac (CARD; n=6) Chagas patients. Top figures: representative t-SNE analysis showing the distribution of the different DN T-cell subpopulations and the location of CD69+ and IL-10+ cells, as indicated. Note the overlap of CD69 and IL-10 expression, and the reduction of CD69+ and IL-10+ cells in CARD. Bottom graphs show the percentage of CD69+IL-10+ DN T-cells expressing TCR alpha-beta or gamma-delta, in the absence (MED) or presence (TRP) of *in vitro* exposure to the parasite for IND and CARD, as indicated. Bars indicate average and standard deviation. Paired and unpaired T tests were used to compare unstimulated and stimulated cultures, or cultures between different group of patients, respectively.

**Supplementary figure 2:** Analysis of IFN-gamma and IL-10 expression by central memory (CM), effector (EF) and naive (N) DN T-cell subpopulations from cardiac Chagas patients (n=4) before and after treatment with anti-CD1d monoclonal antibodies, as described in Material and Methods. Percent frequency of TCR alpha-beta+ or TCR gamma-delta+ CM (A), EF (B) and N (C) DN T-cells expressing IFN-gamma and IL-10 as indicated. The results are expressed as percentage ratio for each culture condition (media+block/media or TRP-SA+block/TRP-SA) in box plots, extending from the 25th to 75th percentile, with a horizontal line at the median with whiskers. Paired T tests were used to compare unstimulated and stimulated cultures.


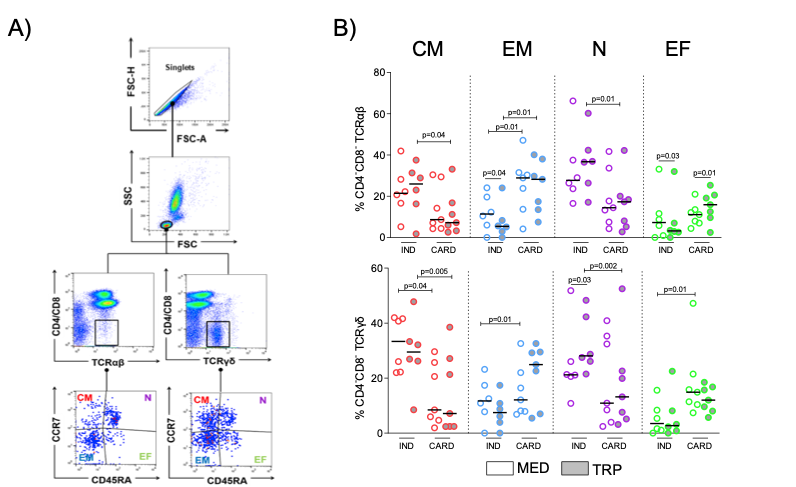


**Supplementary figure 3:** Analysis of DN memory subpopulations, naïve and effector cells in indeterminate (IND, n=6) and cardiac (CARD, n=6) Chagas patients. This is similar to figure 1, with results expressed as individual points, with a horizontal line at the median. A) Representative dot plots illustrating the selection of DN T-cell sub-populations after selection of singlets: central memory (CM), effector memory (EM), naïve (N) and effector cells (EF). Representative plots were performed using data from one IND patient, in non-stimulated culture. B) Frequencies of DN TCR αβ+ or γδ+ CM, EM, N and EF cells before (MED) and after (TRP) *in vitro* stimulation with the parasite. Paired or unpaired T tests were used to compare unstimulated and stimulated cultures, or cultures between different group of patients, respectively. Statistical significance is indicated in each graph.

**Supplementary figure 4:** Analysis of cytokines expression in DN memory subpopulations from indeterminate (IND, n=6) and cardiac (CARD, n=6) Chagas patients. This is similar to figure 2, with results expressed as individual points, with a horizontal line at the median. A) Representative density color illustrating the distribution of DN T-cell sub-populations, and selection of CM DN T-cells, followed by analysis of single and double-positive IFN-gamma^+^ and/or IL-10^+^ cells. Representative density color were performed using data from one IND patient, in TRP-stimulated culture. B) Frequency of IFN-gamma^+^ or IL-10^+^ in central memory (CM) and effector memory (EM) in DN TCR αβ and DN TCR γδ subpopulations. C) Frequency of cells coexpressing IFN-gamma^+^ and IL-10^+^ in CM and EM in DN TCRαβ and DN TCRγδ subpopulations. Paired or unpaired T tests were used to compare unstimulated and stimulated cultures, or cultures between different group of patients, respectively. Statistical significance is indicated in each graph.

**Supplementary figure 5:** Analysis of activation status of DN T-cell central (CM) and effector (EM) memory subpopulations, naïve (N) and effector (EF) cells in indeterminate (IND, n=6) and cardiac (CARD, n=6) Chagas patients gauged by CD69 expression, before (MED) and after (TRP) *in vitro* stimulation with the parasite. This is similar to Figure 3, with the results expressed as individual points, with a horizontal line at the median. The graphs (A and B top) represent the frequency of activated DN T-cell subpopulations in the different groups and under the distinct conditions. Paired or unpaired T tests were used to compare unstimulated and stimulated cultures, or cultures between different group of patients, respectively. Statistical significance is indicated in each graph. A and B (bottom panels) show representative histograms of the expression of CD69 in the different DN T-cell subpopulations in IND and CARD. Clear curves show CD69 expresion in MED and grey curves show CD69 expression in TRP.

**Supplementary table 1:** List of antibodies employed in flow cytomery analysis

| **Antibody** | **Fluorochrome** | **Clone** |
| --- | --- | --- |
| Anti-human CD4 | PE | A161A1 |
| Anti-human CD8 | PE | SK1 |
| Anti-human TCR alpha-beta | Pacific Blue/BV412 | IP26 |
| Anti-human TCR gamma-delta | Pacific Blue/ BV421 | B1 |
| Anti-human CD69 | PE-Cy7 | FN50 |
| Anti-human CD45RA | FITC | HI100 |
| Anti-human CCR7 | APC-Cy7 | G043H7 |
| Anti-human IL-10 | APC | JES3-9D7 |
| Anti-human IFN-gamma | APC/PE-Cy7 | 4S.B3 |
